# Miniaturized Self‐Resonant Micro Coil Array with A Floating Structure for Wireless Multi‐Channel Transmission

**DOI:** 10.1002/advs.202102944

**Published:** 2021-10-29

**Authors:** Byoung Ok Jun, Han‐Joon Kim, Su Jin Heo, Jonghyeun Kim, Jae Hoon Yang, Seunguk Kim, Kyungtae Kim, Woo‐Cheol Jin, Ji‐Woong Choi, Jae Eun Jang

**Affiliations:** ^1^ Department of Information and Communication Engineering Daegu Gyeongbuk Institute of Science & Technology (DGIST) Daegu 711–873 Korea; ^2^ Department of Electrical and Computer Engineering National University of Singapore Singapore 119077 Singapore

**Keywords:** high Q, impedance matching, micro antenna, wireless power transmission

## Abstract

Micro size antennas have significant merits due to the small size effect, enabling new device concepts. However, the low‐quality factor (Q‐factor), the large size of impedance matching components, and the poor selectivity of the multi‐array design remain challenging issues. To solve these issues, a floating coil structure stacked on a loop micro‐antenna is suggested. Various floating coil designs are prepared with appropriate matching conditions at specific target frequencies, using an easy fabrication process without the need for additional space. A simple one‐loop antenna design shows a higher Q‐factor than other, more complicated designs. The micro‐sized loop antenna with the 80 µm trace width design exhibits the highest Q‐factor, around 31 within 7 GHz. The 8 different floating coil designs result in high‐frequency selectivity from 1 to 7 GHz. The highest selectivity contrast and WPT efficiency are above 7 and around 1%, respectively. Considering the size of the antenna, the efficiency is not low, mainly due to the good matching effect with the high Q‐factor of the floating coil and the loop antenna. This micro‐antenna array concept with high integration density can be applied for advanced wireless neural stimulation or in wireless pixel array concepts in flexible displays.

## Graphical abstract

Hybrid array structures of micro‐antenna and various floating coil structures are studied for wireless applications with high selectivity because the hybrid structure shows the appropriate matching condition at specific target frequencies. The micro‐antenna array concept with high integration density can be applied for advanced wireless neural stimulation, micro‐robot, or wireless pixel array concepts in flexible displays.

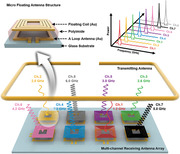

## 1 Introduction

Very small size antennas have been proposed for various new device systems.^[^ ^1^, ^2^, ^3^, ^4^, ^5^, ^6^, ^7^, ^8^, ^9^ ^]^ Such antennas are essential components in certain devices such as microrobots.^[^ ^10^ ^]^ In addition to a single antenna, multi‐array antenna designs can be easily led to various new application concepts because of their small effective size. One good example is the wireless electrode arrays used as neural probes. Recently, there has been considerable interest in developing wireless neural probes for a brain‐machine interface or for brain signal acquisition.^[^ ^11^, ^12^, ^13^, ^14^, ^15^, ^16^, ^17^ ^]^ In the traditional approach, wire connections have been employed to exchange electrical signals between a device and the brain. Unfortunately, the percutaneous cable used to transmit the signal often leaves patients susceptible to infections and can also result in unreliability problems. A more effective alternative method, wireless power transfer (WPT), has emerged, which can supply power or signals to implantable electronics. These implantable electronics have been steadily improved, and WPT technology now plays an important role in modern implants. It provides a remote source of power, which can be wirelessly transferred to the human body. Therefore, such wireless neural stimulation and recording systems can be an essential tool for treating neural disorders and studying biological phenomena.^[^ ^11^, ^12^, ^13^, ^14^, ^15^, ^16^, ^17^, ^18^, ^19^, ^20^ ^]^

But the practical and effective use of such systems in a neural stimulation system, for example, to stimulate neural signals during behavioral modulation, still face significant challenges. A system with high flexibility, lightweight, and smaller size is required that can entirely free‐float on nervous tissue. In recent decades, a significant amount of research effort has been devoted to developing advanced wireless neural stimulators with miniaturized, stretchable, and flexible components to improve their biocompatibility, while maintaining their functionality.^[^ ^14^, ^15^, ^16^, ^17^, ^18^, ^19^, ^20^ ^]^ However, these wirelessly powered neural stimulation systems typically utilize single large size antennas (> ≈cm) and bulky matching components while requiring an implantable battery. The wireless components result in inconveniences and poor biocompatibility even if the issue of wire connection infection is solved. In addition, multi‐channel microelectrode arrays that can stimulate various targeted sites have been widely studied.^[^ ^11^, ^12^, ^13^, ^14^, ^15^ ^]^

**Figure 1:**
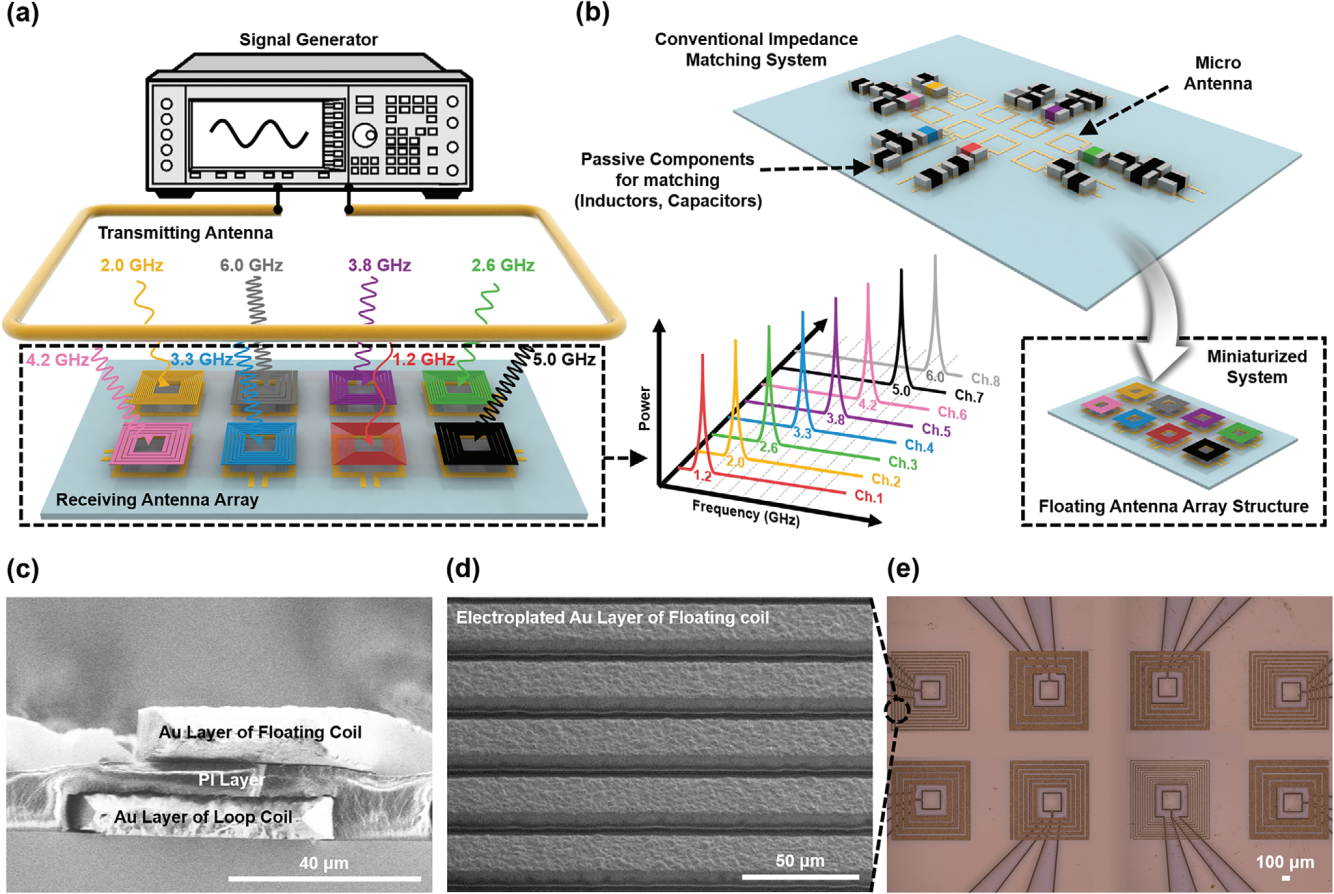
The wireless array system consisting of micro coils floated on loop antennas. a) Conceptual figure for the selective operation of each cell in the array system. b) Conceptual figure of the miniaturized system compared with the conventional impedance matching system. c) SEM cross‐section image of the micro‐coils floated on the loop antennas. d) SEM image of the floating coil. e) OM image of the micro‐coils floated on the loop antennas in the array system.

As a solution, an inductive micro‐sized antenna array has high potential. Due to their small size, each micro antenna in the neural probe with a wireless function performs the role of a single electrode. If each antenna can operate with selectivity and appropriate working efficiency, a simple array structure (**Figure** **1a**) can be a substitute for conventional wires connecting the channels, the need for signal processing chips for the selective transmission of signals to each cell, and a large‐sized antenna. In addition, a modulator to generate neural signals is no longer needed because signal modulation can also be performed from the outside through the on‐off switch.

Herein, we propose a micro antenna array system for potential application in an advanced wireless neural probe. This paper reports the effective enhancement of resistance and Q‐factor, an impedance matching method using a floating antenna structure without complex passive components for the micro‐coil. The selectivity of each antenna is independently operated using different frequencies (1–7 GHz) without a signal processing chip, matching components, or wire connections. This approach can be used to realize various highly simplified and miniaturized systems.

## 2 Results and Discussion

### 2.1 Design of the Floating Antenna Structure in the Array System

Multi‐electrode array designs have been used widely in various applications, such as in the cell design of displays or neural probes. Generally, an electric connection line matrix design or electrical switches such as a transistor are required to drive the multi‐electrode array, and the power or signal is transmitted by wire electrodes. Unfortunately, such systems can be vulnerable to bending, folding, or body implantation. As an alternative approach, if we can develop micro‐scale wireless components, these issues are easily solved. Because each small size antenna can receive signal or power, it can work independently.

Figure 1a shows a schematic diagram of a multi‐micro antenna array used as wireless electrodes. When scaling down the size of the antenna, there is a limit to increasing inductance, and the resistance is significantly increased as the number of turns increases because of the size effect. This results in a degraded quality factor (Q‐factor), an important parameter when attempting to increase WPT efficiency. Moreover, the high resistance makes impedance matching difficult, and the additional lumped elements are required at the desired frequency for the impedance matching.

To enhance the Q‐factor and match the impedance at the desired frequency without the complicated matching components, a floating loop antenna structure with a planar spiral micro‐coil design insulated by polyimide (PI) was developed. (Figure 1b) The 10 µm thick Au layer used for the floating coil and the loop antenna was fabricated using photolithography and electroplating processes. The adhesion of thick Au film on PI was enhanced by O_2_ plasma treatment before the electroplating process.^[^ ^14^ ^]^ If the design is applied to the flexible system, the flexibility can be further enhanced by encapsulating a thick metal layer up and down using polymeric materials. Photo‐sensitive PI was formed between the floating coil and the loop antenna as an insulator. Figure 1c,d shows scanning electron microscope (SEM) images of the fabricated floating antenna structure. Micro‐sized floating antenna structures with different operating frequencies were incorporated in the array system. An optical microscope (OM) image of the fabricated array system is presented in Figure 1e. Using the conventional impedance matching method requires the use of additional passive matching components, wire connections, and electrodes, which results in unintended system expansion and increases parasitic inductance and resistance, which interfere with accurate matching. However, here, since impedance can be matched at the desired target frequency using just the structural effect of the floating antenna structure, without complex matching components, the system can be simplified and miniaturized more effectively.

### 2.2 Frequency Characteristics of Conventional Micro Antenna Structures

**Figure 2:**
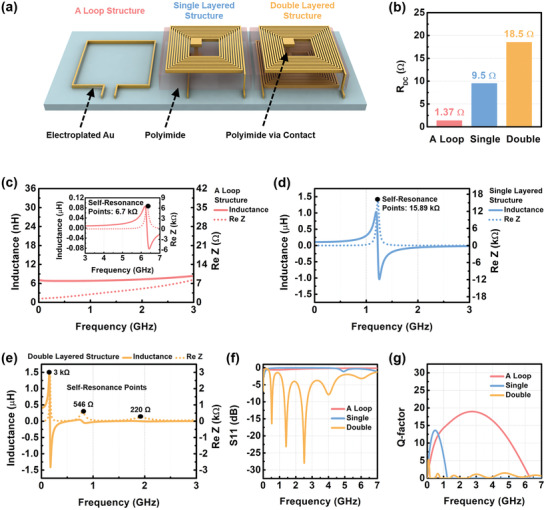
Frequency characteristics of micro spiral coils with conventional structures. a) Conceptual figure and b) DC resistances of the conventional structures of the micro spiral coils. The inductances and the real impedances of c) A loop antenna, d) Single layered structure, and e) Double‐layered structures. f) Scattering parameter S11 and g) Q‐factors according to the structures of the micro spiral coils.

Typically, the inductance of a spiral coil structure can be increased by increasing the number of turns in the macro‐size inductive antenna design, improving the Q‐factor, as well.^[^ ^21^, ^22^ ^]^ Hence, we investigated the frequency characteristics of the system when the number of spiral turns was changed to increase inductance at the micro‐scale. To increase the number of turns, there are some loop micro antenna designs, both with a single layer and a double‐layered structure, as shown in **Figure** **2a**. A loop structure is a 2D structure with 1 turn. The single‐layered structure is composed of one multi‐turn spiral coil with a contact electrode. The double‐layered structure consists of a top planar spiral coil on a PI film insulating layer and a bottom planar spiral coil with a contact electrode. To study the common parameters of the micro antenna structures, a square‐type spiral micro‐coil was adopted. The gold (Au) was deposited using an electroplating process. The measured conductivity was about 11×10^6^ S m^−1^. The trace width and the thickness of the micro‐coils were set to 20 and 10 µm, respectively. Both PI and Au are biocompatible materials, so they are appropriate for an implantable system.^[^ ^23^, ^24^ ^]^ In the single and double‐layered structures, the number of turns (10.5 turns and 21 turns, respectively) is determined by the trace width (20 µm), the spacing (10 µm), and the inner diameter (390 µm). The following fill factor (*d*_o_−*d*_i_ / *d*_o_ + *d*_i_) is about 0.44, where d_o_ and d_i_ are the outer and inner diameters of the coil. The thickness of the PI and bottom electrodes was 13.5 and 10 µm, respectively, with a 3.5 µm gap between the top and bottom electrodes. The frequency characteristics of the prepared square micro antenna structures were analyzed using Ansys HFSS software.

Figure 2b shows the DC resistance resulting from the micro coils structures, which indicates the difference in the number of turns. The DC resistance linearly increased to the length of the structure under the same thickness, as shown in Figure 2b. The double‐layered structure exhibited resistance (18.5 Ω) more than 10 times higher than the loop structure (1.37 Ω) due to the long path. The coil length of the single‐layered structure was about half the length of the double‐layered structure, so that the observed DC resistance was about 9.5 Ω, which is about half the resistance of the double‐layered structure. The DC resistances of the micro‐coil structures were high compared with a macro‐size structure due to the size limit, and this problem can become more serious as the size gets smaller and the thickness of the conductor becomes thinner, depending on the applications. The trace width of the conductor must be reduced to maintain the micro‐coil size as the number of turns is increased. The inductance values of each micro coil structure were extracted from Im(Z)/*ω*, where Im(Z) is the imaginary impedance and *ω* = 2*π*f is the angular frequency, as shown in Figure 2c–e. When the number of turns is increased, from the loop to double‐layered structures, the inductance also increases. The highest inductances (0.086, 1.03, and 1.5 µH) were found at 6.19 GHz, 1.19 GHz, and 155 MHz for the loop, the single, and the double‐layered structure, respectively. However, unlike the resistance value, the inductance of a single structure was not double the inductance of a double‐layered structure. This is because there is a significant increase in parasitic capacitance, creating a large capacitive property in the imaginary impedance, which is produced between turns and additionally forms between the top and bottom layers in the double‐layered structure. The self‐resonance frequency is shifted to a low frequency as the number of turns is increased from a loop structure to the double‐layered structure because the inductance and the parasitic capacitance are increased. After the self‐resonance frequency, the inductive property changes to the capacitive property, so the operating frequency range is lower than the self‐resonance frequency. Therefore, the self‐resonance frequency and the operating frequency are decreased as the number of turns is increased. The highest real impedances of 6.71, 15.89, and 3 kΩ were observed at the self‐resonance frequencies, as shown in Figure 2c–e so that the antenna characteristics are not observed in a loop, and single‐layered structures as shown in Figure 2f and 50 Ω matching adjustment under their self‐resonance frequencies, one of the important parameters for the radiation efficiency of the antenna is required. A loop structure has a wide range of available operating frequencies because the inductive property is maintained up to 6 GHz.

The Q‐factor, the important parameter needed to improve WPT efficiency, was analyzed using the real and the imaginary impedance. The WPT efficiency *η*_12_ can be expressed in Equation (1) by the coupling factor k, and the unloaded Q‐factors of the transmitting and the receiving coils, Q_1_ and Q_2_, respectively, since Q'_2_ = ((Q_2_×Q_L_)/(Q_2_+Q_L_)).^[^ ^21^ ^]^

(1) $$\eta_{12}=\left(\frac{k^{2}Q_{1}{Q^{\prime }}_{2}}{1+k^{2}Q_{1}{Q^{\prime }}_{2}}\right)\left(\frac{Q_{2}}{Q_{2}+Q_{L}}\right)$$

Hence, the improvement in Q‐factor results in a higher WPT efficiency. Generally, the Q‐factor is defined as *ω*L/R, which can be improved by increasing the inductance L and decreasing the parasitic AC resistance R. Accordingly, the Q‐factor of the micro antenna structures are calculated from Im(Z)/Re(Z), where Re(Z) is the real impedance. However, increasing the number of turns to enhance the inductance leads to a serious increase in resistance due to the geometric effect of the micro antenna. The high Q‐factor of micro‐sized antennas is known to be in the GHz range, so it is advantageous to use an operating frequency in this range. However, the resistance rapidly increases in the GHz range, so the resistance problem becomes more serious due to the skin effect.^[^ ^25^ ^]^ As a trade‐off between the inductance and the resistance, the one‐loop design shows the highest Q‐factor, as shown in Figure 2g, even though it has the lowest inductance (Figure 2c). The change in resistance is not considerable at the macro level as the total length of the antenna increases to a certain value since the antenna commonly consists of thick copper wire. Accordingly, the Q factor tends to improve as the inductance increases, while the change in resistance at the micro‐level is significant. Hence, unlike at the macro level, resistance affects the Q‐factor value more at the micro size level than the inductance. In the single and double‐layered structures, the Q‐factors were fairly lower than that of a loop structure, within 6 GHz. Thus, among the three structures, a loop structure with the highest Q‐factor is the appropriate structure for a micro‐scale WPT system. Moreover, the self‐resonance frequency was observed to be near 6 GHz, and this wide range allows the design of antennas with varying specific operating frequencies, achieved through impedance matching.

### 2.3 Frequency Characteristics of the Floating Antenna Structure

Considering the previous results, it is clear that reducing resistance can be more effective than increasing inductance to improve the Q‐factor and that the loop antenna is appropriate for WPT at the micro‐level. It is also necessary to induce a real impedance close to 50 Ω at the resonance point for impedance matching with the additional passive components. In our concept, it is important to keep the size of the micro‐size antenna small and retain biocompatibility. Using traditional bulky matching components in our system is not appropriate because the sizes of the components are generally larger than the micro antenna. Furthermore, the passive components include lots of parasitic parameters like inductance, capacitance, and resistance. In addition, the contact lines and electrodes needed to connect the coil and matching components contain a number of unwanted parasitic values as well. These parasitic values can be negligible at the macro‐level, but fatal at the micro‐level since the micro antenna, matching components, contact lines, and electrodes are all at the same micro‐level. Furthermore, a small change of size can result in a large change in frequency characteristics since the operating frequency is above GHz. In particular, an increase in parasitic resistance significantly degrades the Q‐factor. This makes it necessary to avoid direct connections and prevent further increases in parasitic resistance for impedance matching without additional passive components.

**Figure 3:**
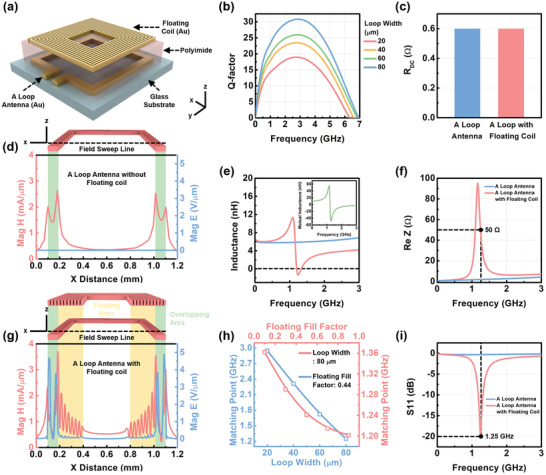
Structure of the micro‐coil floated on the loop antenna. a) Conceptual figure of the micro‐coil floated on the loop antenna. b) Q‐factors according to the trace widths of the loop antennas. c) DC resistance comparison of loop antennas with and without the floating coil. d) H‐field and E‐field of a loop antenna without the floating coil. e) Inductance comparison of loop antennas with and without the floating coil and the mutual inductance. f) Real impedance comparison of loop antennas with and without the floating coil. g) E‐field and H‐field of a loop antenna with the floating coil. h) Matching point variations according to the fill factor of the floating coil and the trace width of the loop antenna. i) Scattering parameters S11 comparison of loop antennas with and without the floating coil.

Here we propose a floating antenna structure with a high Q‐factor and impedance matching without complex matching components, contact lines, or electrodes. This design results in a simplified and miniaturized system. **Figure** **3a** depicts the floating antenna structure. The top planar spiral coil with multi‐turns is floated on the bottom micro loop antenna by PI film. The Q‐factor of the loop antenna can be further improved by increasing the trace width and decreasing the resistance of the conductor, as shown in Figure 3b. The chosen trace width of the loop antenna and the floating coil line were 80 µm and 20 µm, respectively. The fill factor was fixed at 0.44, the same as the previous condition. The following DC resistance of the loop antenna with the floating coil was 0.6 Ω, the same as the resistance of the loop antenna without the floating coil shown in Figure 3c because the floating coil is not directly connected to the loop antenna.

The floating coil and the loop antenna are indirectly coupled by E‐field and H‐field, as shown in Figure 3d, g. At first, the magnetic field is formed around the loop antenna wires, as shown in Figure 3d. The magnetic field is also formed around the floating antenna wires. It is shown that the magnetic field is strongly formed at the spacing between the turns because the wire is adjacent to the next wire, and the magnetic field is reinforced, as shown in Figure 3g. The addition of the floating coil on the loop antenna results in further reinforcement of the magnetic field between two antennas. Around the area that the wires of floating and loop antennas are overlapped (overlapping area: green part in Figure 3g), the magnetic field is strongly formed. At the floating area (yellow part in Figure 3g) close to the traces of the loop antenna, the magnetic field is relatively more reinforced and increased. On the contrary, the magnetic field is decreased at the area close to the center of the loop antenna due to the reinforcement reduction. The electric field is also investigated. Compared with the electric field without the floating coil, as shown in Figure 3d, the electric field is strongly formed at the overlapping area where metal traces face each other, as shown in Figure 3g. One of the specific points is that the inductance is increased by coupling with mutual inductance, as shown in Figure 3e. The mutual inductance can be found by summing the partial mutual inductance values between one turn on a loop antenna and all turns on the floated coil.^[^ ^26^, ^27^ ^]^ Using the Maxwell equations, the mutual inductance *M_ij_* between a pair of parallel circular single‐turn coils at radius *r_i_* and *r_j_* for perfectly aligned coaxial coils can be expressed in Equation (2).

(2) $$\begin{array}{ccc} & & M_{ij}=\frac{2\mu }{\alpha }\sqrt{r_{i}\cdot r_{j}}\left[\left(1-\frac{\alpha^{2}}{2}\right)K\left(\alpha \right)-E\left(\alpha \right)\right]\\ & & \alpha =2\sqrt{\frac{r_{i}\cdot r_{j}}{{\left(r_{i}\cdot r_{j}\right)}^{2}+D^{2}}} \end{array}$$

Where D is the relative distance between the two coils, μ is the permeability of the medium, *K*(*α*) and *E*(*α*) are the complete elliptic integrals of the first and second kind, respectively. Equation (2) indicates that the mutual inductance from the relative distance between two coils is adjustable as the thickness of the PI varies. For the PI gap (1 µm), the real impedance can achieve 50 Ω at 1.25 GHz near the self‐resonance frequency, as shown in Figure 3f. Moreover, the matching frequency is adjustable by varying the fill factor of the floating coil at the fixed trace width of the loop antenna (80 µm), and changing the trace width of the loop antenna with the static fill factor of the floating coil (0.44), as shown in Figure 3h. The scattering parameter S11 changes up to about −20 dB at 1.25 GHz, which proves that the impedance is well‐matched to 50 Ω at the target frequency, as shown in Figure 3i. In other words, the floating coil enables impedance matching at the desired frequency without an increase in parasitic DC resistance. This impedance matching approach can simplify a 3D bulk structure into a 2D structure and effectively reduce the overall size to a micro‐level. This effect is more pronounced in the array structure, which enables a highly miniaturized and flexible system, as shown in Figure 1b.

### 2.4 The Details of Impedance Matching Using the Geometric Values of the Micro Floating Coil Structure

**Figure 4:**
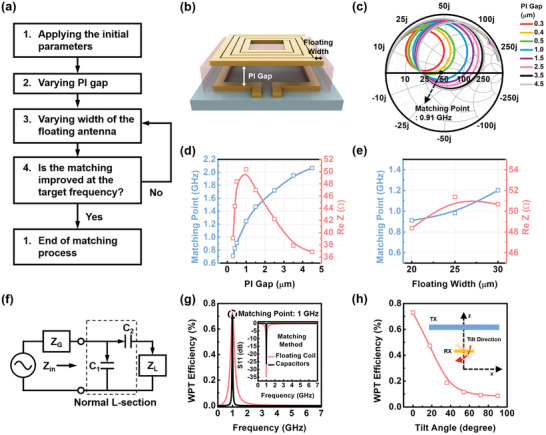
Impedance matching process using structural parameters of the micro‐coils floated on the loop antennas. a) Block diagram of the impedance matching process while varying the PI gap and the trace width of the floating coil. b) Structural parameters in the micro coils floated on the loop antennas. c) Smith chart according to the variation in PI gap. d) Matching point and real impedance with the varying PI gap. e) Matching point and real impedance while varying the trace width of the floating coil. f) Equivalent circuit for the conventional impedance matching method. g) Comparison of WPT efficiency using the conventional matching method and floating coil. h) WPT efficiency according to the misalignment angle between TX and RX.

Additional details about the floating antenna structure were investigated for impedance matching. In this structure, some parameters (the trace width, the spacing of the floating coil, and PI gap) can change the E‐field and the H‐field. Thus, varying the parameters allows us to adjust impedance matching for the target frequency. Here, an impedance matching method at the desired frequency is suggested while varying the parameters in the floating antenna structure. **Figure** **4a** shows the process for optimizing the impedance matching. It begins with a set of initial values and ends with the optimal geometries of the floating antenna structure at the predetermined frequency. HFSS simulations were used for the optimization process.

The matching process starts with the initial parameters of the matching frequency near a target matching point. In step 1, the target matching frequency and the minimum parameters are determined by fabrication limit. Here, we set the target frequency (1 GHz), the conductor thickness (10 µm), the fill factor (0.44), the trace width of the micro loop antenna (80 µm), and the spacing between conductor traces of the floating micro coil (10 µm). The trace width of the floating coil is one of the factors that can easily shift the matching frequency across a wide range because a large change in trace width can cause a large change in the resonance frequency, and changing the trace width is not difficult in the fabrication step. Hence, it is necessary to set the initial trace width and bring it near the desired frequency.

When the initial trace width (20 µm) was determined, the initial matching frequency and real impedance were found at 2 GHz and about 37 Ω, respectively, with 4.5 µm of PI thickness. The matching frequency is required to move to the target frequency of 1 GHz. By varying the PI gap in step 2, the matching point can be moved near the target frequency, as shown in Figure 4c,d. The corresponding S11 parameters are shown in Figure S1a, Supporting Information. The matching point is shifted to the lower frequency level as the PI gap is decreased by up to 300 nm with respect to the initial matching point. At 500 nm of the PI gap, the matching frequency was obtained at 0.91 GHz, and the following real impedance was about 48 Ω, respectively. However, our target frequency was 1 GHz, so the next step is required to finely adjust the matching frequency. Of course, if the PI thickness becomes a little bit thicker, we may get exact matching of the 1 GHz target frequency. However, the thickness was controlled by spin‐coating speed, and the process margin was not big. Instead, fine‐tuning the trace width was easy because we used photolithography to control the trace width.

In step 3, the PI gap of 0.5 µm with the closest matching frequency to 1 GHz was fixed, the trace width of the floating coil was changed once more with a small difference, from 20 to 30 µm as shown in Figure 4e. The corresponding S11 parameters are attached to Figure S1b, Supporting Information. As a result, we found the optimal parameters (a PI gap of 0.5 µm and a floating coil trace width of 25 µm) for the impedance matching at 1 GHz. The real impedance was adjacent at 50 Ω. When the floating antenna structure from step 3 improves the impedance matching at the desired frequency, the matching process is ended. However, impedance matching can be further enhanced by iterating through steps 2–3, and the matching process can continue until the improvement ends.

To verify our matching method, the final matching results were analyzed by comparison with the conventional matching method.^[^ ^28^, ^29^ ^]^ Using the normal L‐section impedance matching method, the ideal capacitors excluding parasitic inductance and resistance were connected in series and in parallel to compare with our impedance matching method, as shown in Figure 4f. The capacitors C_1_(24 pF) and C_2_(5.5 pF) were connected in parallel and in series, respectively, with the loop antenna (trace width: 80µm). In practice, the capacitors include parasitic inductance and resistance. Moreover, it is not only difficult to meet the exact capacitance but it is also necessary to connect more capacitors in series or in parallel to meet the value. Additionally, the necessary contact pads and contact lines are also needed. The result is the parasitic parameters are uncontrollably increased, and the size of the matching system is significantly expanded.

Even though our matching system was compact in size, with a simple matching method, the results showed it had performance comparable to the ideal case of conventional matching methods, as shown in Figure 4g (inset).

To verify WPT efficiency using conventional and our matching methods, the transmitting antenna (TX) was prepared with a 6 × 3 mm outer diameter and 0.5 mm Cu thickness. The WPT efficiencies of the conventional and our matching methods were almost identical, as shown in Figure 4g when the TX and each receiving antenna (RX) had a distance of 1 mm. This result shows that our matching method is simple and more advanced, with a compact size. Additionally, the availability of our antenna design as an implantable unit is investigated at the tissue condition. At the condition with the antenna covered in the two‐layer human tissue model as shown in Figure S2a, Supporting Information, WPT efficiency is verified as shown in Figure S2b, Supporting Information. The electrical property in the different human tissue, where the conductivity and the relative permittivity of skin and fat are 0.9 S m^−1^, 0.054 S m^−1^, 41, and 5.45, respectively, is studied at 1 GHz of the matching frequency. Compared with WPT efficiency in an air gap and in the tissue, the matching frequency in the tissue shifts by ≈ 0.1 GHz from the air gap, and the efficiency decreases by 0.01%. Therefore, our antenna design is appropriate to be used as an implantable unit. Angular orientation may occur if the antenna is implanted into the human body. The WPT efficiency is further investigated according to the angular orientation changes, as shown in Figure 4h. WPT is quite sensitive to angular orientation. At the 30 degrees condition, the efficiency is 50% of the parallel condition. The efficiency can be improved by TX antenna design.^[^ ^30^, ^31^ ^]^

### 2.5 Frequency Characteristics in the Array System with the Floating Antenna Structure

**Figure 5:**
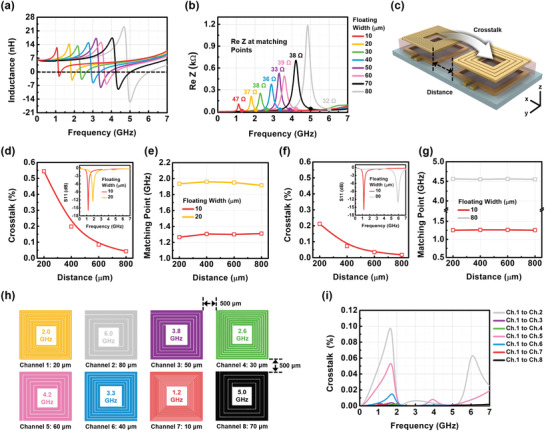
Frequency characteristics of the micro‐coils floated on the loop antennas in the array system. a) Inductance and b) Real impedance depending on the trace width of the floating coil. d) Crosstalk and e) Matching point variation of the floating antenna structure (floating trace width: 10 µm) when another floating antenna structure (floating trace width: 20 µm) with a similar matching frequency was close to the side. f) Variation in crosstalk and g) Matching point of the floating antenna structure (floating trace width: 10 µm) when another floating antenna structure (floating trace width: 80 µm) with a large difference in matching frequency was close to the side. h) Arrangement of the floating antenna structure in the array system. i) Crosstalk from channels 2–8 to channel 1 in the array system.

From the previous results, by adjusting various parameters, we found that a floating antenna structure could be designed with the desired operating frequency. Next, micro floating antenna structures with different operating frequencies were formed into an array system using various trace widths of floating coils, and their frequency characteristics were analyzed. The 8 same loop antennas with trace widths of 80 µm were arranged in 4 × 2 design (≈channel 1–channel 8). Floating coils with different designs (trace widths: ≈10–80 µm) using different turns of the antenna were placed on a loop antenna with a 3.5 µm thick PI gap. Different ≈nH levels of inductance were obtained depending on the trace widths of the floating coils, and each antenna structure had a different operating frequency, as shown in **Figure** **5a**. Furthermore, we confirmed it was possible to independently drive frequencies in the array system since the matching points observed near each resonant point were separated by enough resolution, as shown in Figure 5b. As the trace width of the floating coil in the floating antenna structure grew wider, higher matching frequencies were obtained. The impedance matching could be further enhanced using the matching process described earlier.

To apply the floating antenna structures with 8 different matching frequencies to an array system, crosstalk needs to be considered, which involves power loss transferred from one channel to another.^[^ ^32^, ^33^, ^34^ ^]^ This is because the power transferred from TX to the target site in the RX array can be transmitted to other undesired sites. This makes it important to reduce crosstalk because crosstalk by adjacent coils reduces WPT efficiency. Crosstalk is determined by the wireless power efficiency transferred from one channel to other and depends on the distance from the side coil, and the difference in matching frequency, as shown in Figure 5c. If the matching frequencies of two floating antenna structures (e.g., the trace widths of the floating coils: 10 and 20 µm) are adjacent (Figure 5d (inset)), the distance separating the two side coils is important. When two antenna structures are closer, the crosstalk is higher, as shown in Figure 5d. The highest crosstalk was 0.55% at 200 µm of separated distance and could be reduced down to 0.04% by increasing the separation distance (800 µm). If the matching frequency is changed based on the separation distance between the two side coils, additional impedance matching is required. Hence, we investigated changes in frequency according to the separated distances. Even if there are slight changes of matching points, the frequency change was negligible because they change a little, and it does not significantly affect independent driving in the array, as shown in Figure 5e.

However, there is a limit to how wide the separated distance can be increased since it expands the size of the whole system. As an alternative solution, Figure 5f shows another way to reduce crosstalk, using an arrangement of antenna positions, that is, two coils with large differences of matching frequencies (the trace widths of the floating coils: 10 and 80 µm) were positioned close to each other, as shown in Figure 5f (inset). The crosstalk of the arrangement was 0.21% at the separation distance of 200 µm and 0.017% at the separation distance of 800 µm. Moreover, there was no change in matching frequency with the separated distances, as shown in Figure 5g. Accordingly, floating antennas with similar designs and small differences in matching frequency are better placed as far away as possible from each other in the multi‐antenna array system, as shown in Figure 5h.

After optimizing the placement of the 8 floating antenna structures in the array system, the transferred power efficiency from channel 1 to the other channels (channel 2–8) was analyzed, and the crosstalk was maintained to less than 0.1%, as shown in Figure 5i. As a result, the integrated array system was able to selectively drive 8 cells with floating antenna structures using 8 different frequencies (≈1–7 GHz) without complex matching components, contact pads, or wire connections.

### 2.6 Wireless Selective Driving of the Micro Floating Coils in the Array System

**Figure 6:**
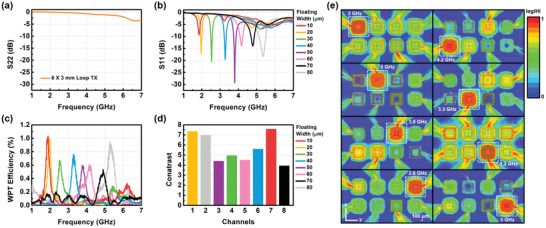
Selective operation of each cell in the array system using WPT with different operating frequencies. a) Scattering parameter S22 of TX. b) Scattering parameter S11 of the micro‐coils floated on the loop antennas in the RX array system. c) WPT efficiencies from TX to the micro‐coils floated on the loop antennas in the RX array system. d) Contrast comparison at each channel. e) H‐fields of the micro‐coils floated on the loop antennas in the RX array system while transmitting wireless power from the TX.

We confirmed the frequency characteristics of the floating antenna structures in the array system. Here, the characteristics of WPT were demonstrated to selectively drive each cell using different operating frequencies. Based on the arrangement in Figure 5f, the micro floating antenna structures with different trace widths of floating coils were prepared in an array system. **Figure** **6d** represents the 8 different fabricated micro floating coils (trace widths of the floating coils: 10 to 80 µm) on the same loop antenna design (the trace widths of the loop antennas: 80 µm) with a 3.5 µm thick insulating PI gap between the floating and loop antennas (conductor thicknesses: 10 µm) in the array system. Our array system has a very small size compared to cables and connectors for the measurement. Due to the relative size difference, unwanted parasitic parameters from these cables and connectors are too large and need to be eliminated to verify the exact frequency characteristics of the array system. Therefore, we chose the network analyzer to demonstrate the frequency characteristics of the floating antenna structure because the network analyzer can calibrate undesired frequency characteristics generated from cables and connectors for measurement. A TX with a 6 × 3 mm outer diameter and 0.5 mm Cu wire was designed to be able to cover the entire RX array for WPT. Then, the scattering parameters S11 and WPT efficiency were measured using a network analyzer. To selectively drive RX, the TX was designed without matching points within 7 GHz, as shown in Figure 6a. Therefore, the operating frequencies for WPT are highly dependent on the matching frequencies in the RX.

The WPT efficiency is obtained by converting S21, as shown in Equation (3).

(3) $$\eta_{21}=100\cdot 10^{\left(S21/10\right)}$$

The matching frequencies in the RX were differentiated using the design parameters of the floating coils in the array system, as shown in Figure 6b. Wireless power was selectively transmitted with high efficiency at each matching frequency (Figure 6c). The highest WPT efficiency ≈1% was observed in channel 1 (floating trace width: 20 µm) and the lowest efficiency of 0.53% was found in channel 8 (floating trace width: 80 µm). The average WPT efficiency of the 8 channels was about 0.77%. Considering the size of the antenna, this efficiency is not low, mainly due to the good matching effect with high Q‐factor from the floating and the loop antenna. We confirmed that each cell could be selectively driven at the desired frequency. The WPT efficiency can be improved by matching the frequencies of the TX corresponding to the RX matching frequencies by connecting variable capacitors to the TX, as shown in Figure S3, Supporting Information. There was a slight error in the measurement results caused by the fabrication margin of the PI film between the floating and the loop antennas in each cell of the array system.

In the impedance matching process shown earlier, the matching frequency changes significantly depending on the PI thickness. When the PI is formed on the loop antenna, the top surface is not flat because of the thick Au film of the loop antenna structure, unlike the ideal simulation design. The effect of the bump structure of PI film is considered as shown in Figure S4a, Supporting Information. With increasing the height of the bump structure, the matching frequency shifts to a higher frequency. For the bump structure (3 µm) of PI film, the matching frequency is shifted from 1.25 to 1.8 GHz. Hence, a planarization process by a chemical mechanical polishing (CMP) process can be a solution to minimize error. We believe that the CMP process will enable accurate results in future works.

The strength of the H‐field was simulated by HFSS at the operating frequencies of each cell in the RX array system. Wireless power was transmitted to the RX from the TX as shown in Figure 6e. It was visually confirmed that the H‐field was strongly generated at the operating frequencies of each target antenna cell in the RX array system, equivalent to the frequencies where the WPT efficiency appeared higher. The average contrasts between a target cell and other cells were calculated using the data in Figure 6c, as shown in Figure 6f. The maximum contrast value of 7.59 was observed for channel 7, and the lowest contrast was about 4 in channel 8. The average contrast of each channel was about 5.67, providing sufficient contrast for selective driving.

Based on these results, we confirmed that an RX array system with micro coils floated on the loop antennas could be realized. These highly simplified and miniaturized systems can be used as flexible and implantable systems for advanced wireless neural stimulation, with multi‐functions driven by different frequencies without additional components.

## 3 Conclusion

The importance of simplified and miniaturized wireless systems with multi‐function will continue to increase. In this study, we developed a highly simplified and miniaturized RX array system (5.5 × 2.5 mm) with 8 different micro coils floated on loop antennas. The array is selectively driven using 8 different operating frequencies to demonstrate their potential application in highly stretchable and flexible systems with multi‐functions. To enhance the Q‐factor of the micro antenna, the impedance at the desired frequency was matched without the use of complex matching components. By simplifying the RX array system, the structure of the micro‐coil could be floated on the loop antenna. An impedance matching method was proposed that used the structural parameters without additional matching components. We proved that our matching method was more simple and accurate than the conventional impedance matching method. Then, to reduce the crosstalk between the side antennas in the array system, the antennas with large operating frequency differences were arranged, and the array system was constructed with appropriate distances between the side antennas. Finally, we verified that each cell in the fabricated array system could be selectively driven at the different operating frequencies. This approach provides a new direction for achieving a highly stretchable, multi‐function, and flexible system for advanced wireless neural stimulation.

## 4 Experimental Section

### Fabrication of the Micro Coils Floated on the Loop Antennas in the Array System

In the array system, 8 loop antennas with trace widths of 80 µm were placed on a glass substrate, and the 8 types of multi‐turn coils were floated on each loop antenna with a PI insulation gap. The resistance problem becomes serious as the antenna becomes micro‐scale. Increasing the number of turns to meet the desired frequency characteristics resulted in a significant increase in resistance. As increasing the size in two dimensions brings the limitations of the desired application, the best way to reduce resistance was to increase thickness in three dimensions. Sputtering and evaporative systems were limited when depositing thick metal patterns and can only provide thicknesses under 1 µm. Hence, the electrodeposition process was used to increase the thickness up to 10 µm. However, the electrodeposition system provides poor metal crystallization compared to the sputtering and the evaporation systems. This can be improved by controlling the plating current, temperature, and circulation of the plating solution, as shown in Figure S5, Supporting Information. The fabrication of the floated antenna structure was performed as follows (Figure S6, Supporting Information). A seed layer of 50 and 100 nm thick chrome (Cr) and gold (Au), respectively, were deposited on the front of a 4‐in boro‐glass wafer using a radio frequency (RF) sputtering system. The photoresist (JSR THB‐126N) was patterned using the photolithography process. Alkaline cyanide deposition was used for Au electroplating, in which the electrolyte was based on the potassium dicyanoaurate (K[Au(CN)_2_]). 10 µm thick Au was electroplated at 30 °C and 0.03 A for 2 h in 20‐liter reserve bath of the electroplating machine to fabricate a loop antenna, and the following flow of the plating solution using a magnetic pump was about 30 l m^−1^. However, there is the possibility that electroplating Au was peeled off from the glass substrate because of the high aspect ratio patterns. Hence, Cr adhesion and Au seed layers were deposited on O_2_ plasma‐treated glass substrate to improve the adhesion between electroplating Au of the loop antenna and glass substrate.^[^ ^35^ ^]^ The photoresist was removed by dipping in the STR‐F stripper at 60 °C. The Cr / Au seed layers (50 nm / 100 nm) were etched using an inductively coupled plasma (ICP) reactive ion etching (RIE) system with Cl_2_ gas. The photosensitive PI was spin‐coated, then patterned using the photolithography process, cured at 190 °C for 1 h, and treated by O_2_ plasma at 100 W to improve the adhesion between electroplating Au of the floating coil and PI film. Then, the floated antenna was fabricated using the same process before patterning the PI. Figure 1c–e presents OM and SEM images of the fabricated array system with the floated micro antennas.

### Characterization and Measurement of the Floating Antenna Structures in the Array System

Modeling the floating antenna structures and simulation of their frequency characteristics (S‐parameters, impedances, electric field distributions) were conducted using ANSYS HFSS in ANSYS Electronics Desktop 2020 R2 (ANSYS Corp., Canonsburg, PA). Using the normal L‐section impedance matching method, the frequency characteristics of the loop antenna modeled in HFSS and connected with the capacitors in series and in parallel were analyzed in cooperation with ANSYS Circuit in ANSYS Electronics Desktop 2020 R2. To demonstrate the frequency characteristics of the floating antenna structures, each cell in the fabricated array system was connected with SMA connectors (frequency range: 0–12.4 GHz) and the frequency characteristics were measured by connecting to the network analyzer N5247A PNA‐X (Keysight Technologies, Inc.). Then, to verify the selective driving of each cell using WPT with different operating frequencies, TX was connected to port 2, and wirelessly transferred powers to each cell in RX array system connected to port 1 were measured using the network analyzer.

## Conflict of Interest

The authors declare no conflict of interest.

## Supporting information

Supporting Information

## Data Availability

Research data are not shared.
