# Supplementary material for: Miniaturized Self‐Resonant Micro Coil Array with A Floating Structure for Wireless Multi‐Channel Transmission
Source: Adv Sci (Weinh). 2021 Oct 29;8(24):2102944. doi: 10.1002/advs.202102944 (PMC8693062; doi:10.1002/advs.202102944)
Supplement: Supplementary file 1 — Supporting Information [file ADVS-8-2102944-s001.pdf]

© 2021 Wiley-VCH GmbH

ADVANCED SCIENCE
Open Access

Supporting Information

for *Adv. Sci.,* DOI: 10.1002/advs.202102944

Miniaturized self-resonant micro coil array with a floating structure for wireless multi-channel transmission

*Byoung Ok Jun, Han-Joon Kim, Su Jin Heo, Jonghyeun Kim, Jae Hoon Yang, Seunguk Kim, Kyungtae Kim, Woo-Cheol Jin, Ji-Woong Choi, and Jae Eun Jang**

Supporting Information

Figure S1. Impedance matching process using structural parameters in the micro coils floated on the loop antennas.

Figure S2. WPT efficiency in tissue after impedance matching using floating coil.

Figure S3. Selective operation of each cell in the array system using the impedance matched TX at the operating frequencies corresponding to each cell.

Figure S4. The frequency characteristic according to the planarization of PI film.

Figure S5. Resistance variation according to the electroplating conditions.

Figure S6. Fabrication process of the floated antenna structures in the array system.

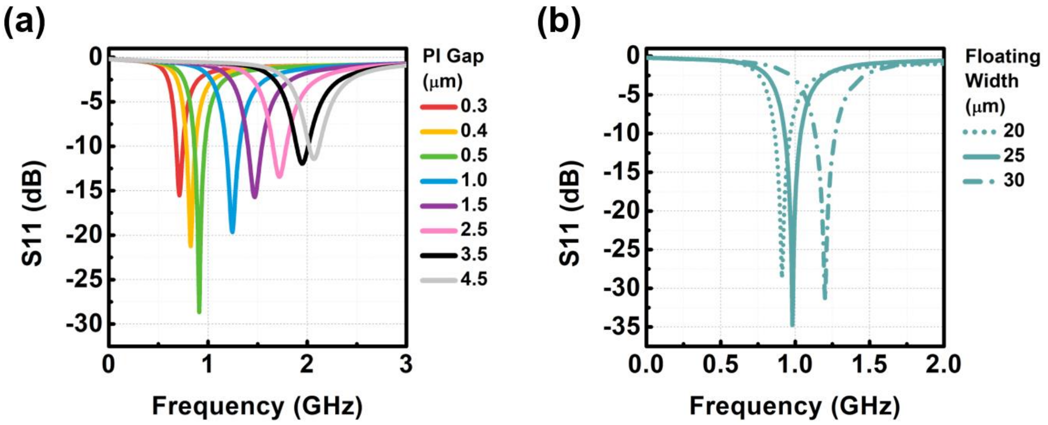

Figure S1. Impedance matching process using structural parameters in the micro coils floated on the loop antennas. (a) Scattering parameter S11 variation as varying the PI gap. (e) Scattering parameter S11 variation as varying the width of the floating coil.

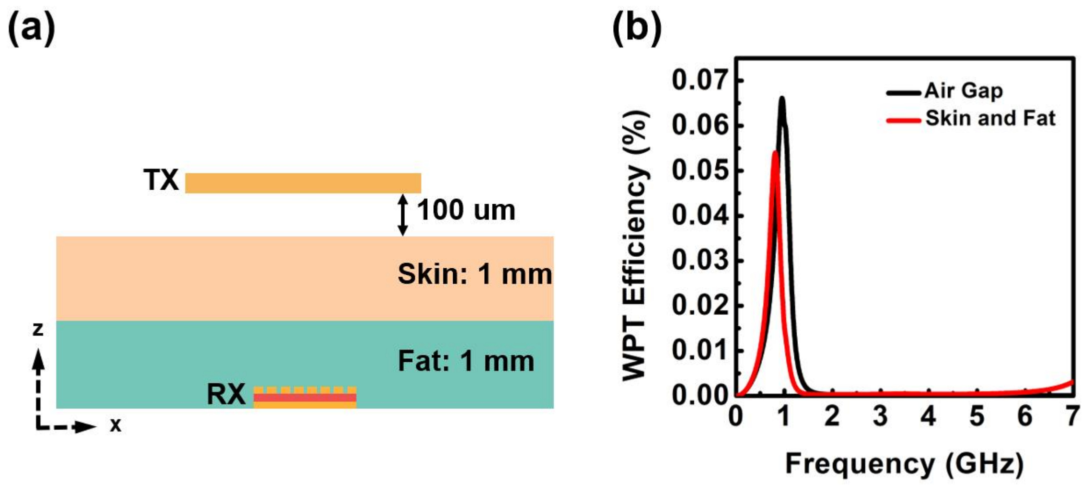

Figure S2. WPT efficiency in tissue after impedance matching using the floating coil. (a) Schematic figure of the two-layer tissue (skin and fat) model in HFSS. (b) WPT efficiency in the air gap and in the tissue.

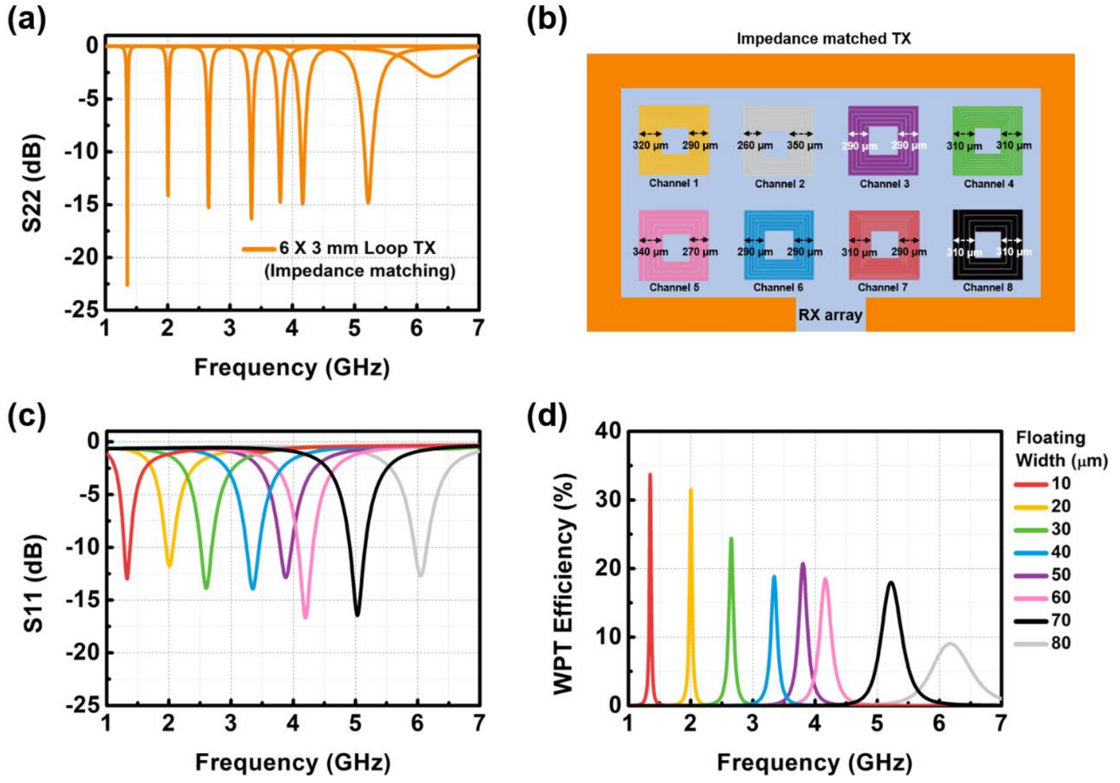

Figure S3. Selective operation of each cell in the array system using the impedance matched TX at the operating frequencies corresponding to each cell. (a) Scattering parameter S22 of the impedance matched TX. (b) WPT system using the impedance matched TX. (c) Scattering parameter S11 of the micro coils floated on the loop antennas in the RX array system. (d) WPT efficiencies from the impedance matched TX to the micro coils floated on the loop antennas in the RX array system.

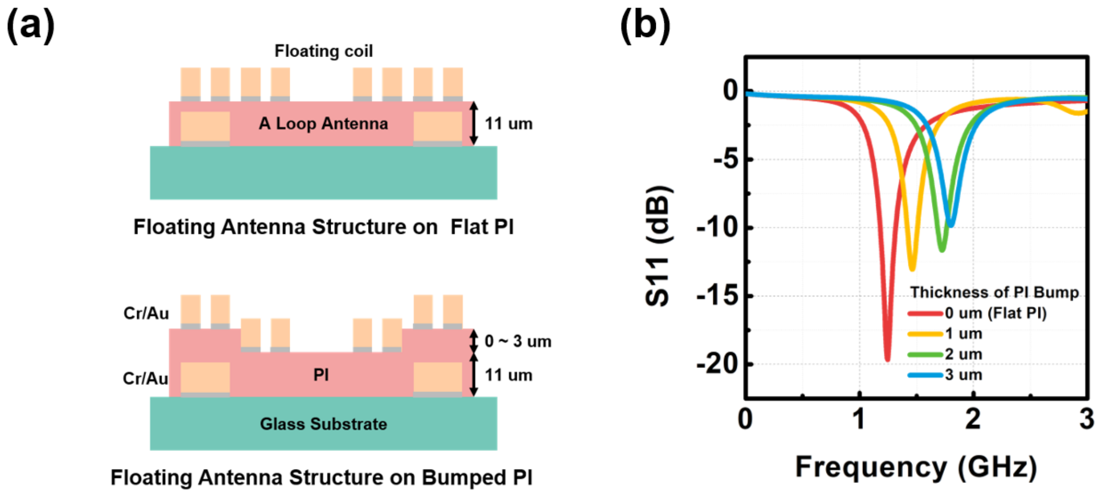

Figure S4. The frequency characteristic according to the planarization of PI film. (a) Schematic figure of the floating antenna structures on the flat PI and on the bumped PI structures (The trace width of the floating coil: 20 um, the trace width of a loop antenna: 80 um). (b) S11 parameters of the floating antenna structures on the flat PI and on the bumped PI structures.

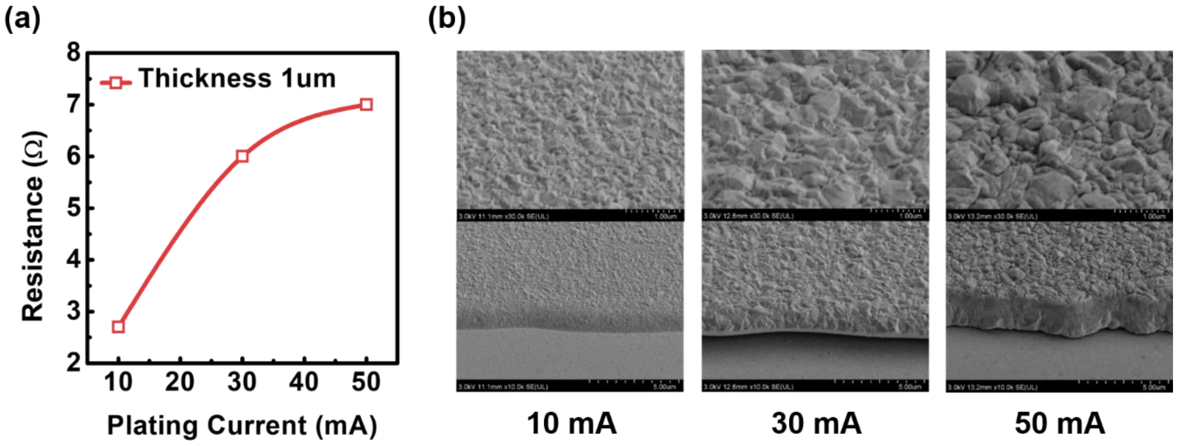

Figure S5. Resistance variation according to the electroplating conditions. (a) Resistance of thin film as varying the electroplating current. (b) SEM images of electroplated Au according to the electroplating current.

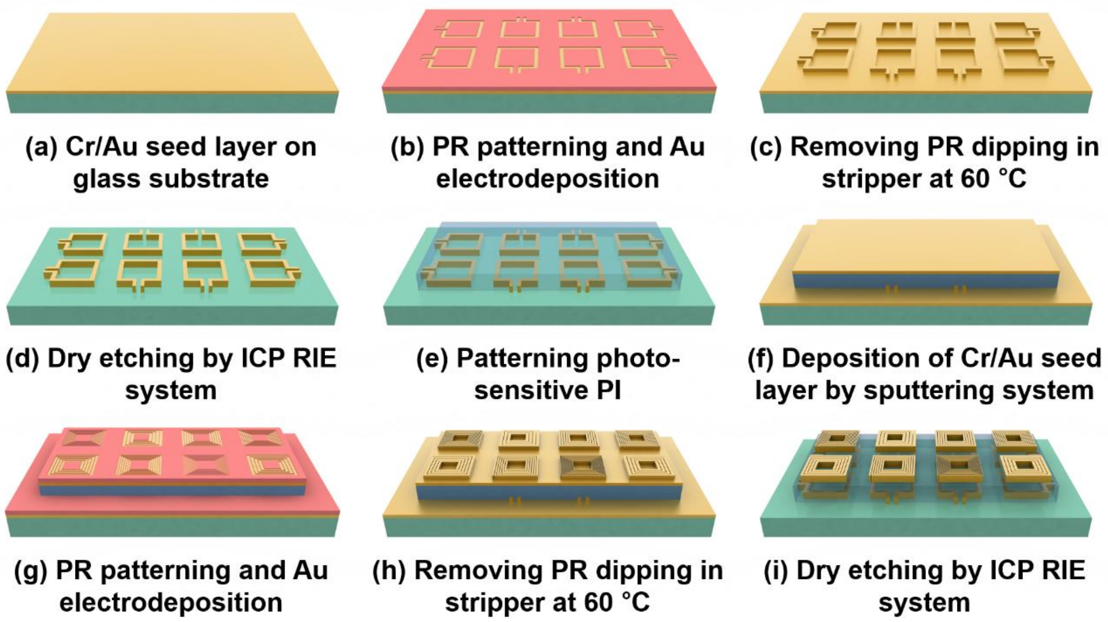

Figure S6. Fabrication process of the floated antenna structures in the array system.
